# In vitro study of foot bone kinematics via a custom-made cadaveric gait simulator

**DOI:** 10.1186/s13018-020-01830-3

**Published:** 2020-08-24

**Authors:** Genrui Zhu, Zhifeng Wang, Chengjie Yuan, Xiang Geng, Jian Yu, Chao Zhang, Jiazhang Huang, Xu Wang, Xin Ma

**Affiliations:** Department of Orthopedics, Huashan Hospital, Fudan University, No.12, Middle Wulumuqi Road, Jingan District, Shanghai, China

**Keywords:** Cadaveric gait simulator, In vitro kinematics, Foot bones, Gait

## Abstract

**Background:**

Quantifying detailed kinematics of the intrinsic foot bone during gait is crucial for understanding biomechanical functions of the foot complex musculoskeletal structure and making appropriate surgery decisions.

**Research question:**

The purpose of this experiment is to measure bone kinematic of the normal foot in a gait cycle via a custom-made cadaveric gait simulator.

**Methods:**

In this experiment, we used a custom-made 6 degrees of freedom (DOF) of robotic gait simulator simulating normal human gait to measure the 3-dimensional (3D) kinematics of tibia, calcaneus, cuboid, navicular, medial cuneiform, first metatarsal, and fifth metatarsal through six cadaveric feet.

**Results:**

The results showed that the kinematic of the intrinsic foot bones in the stance phase of the gait was successfully quantified using a custom-made robotic gait simulator. During walking stance, the joints in the medial column of foot had less movement than those in the lateral column. And during the later portion of stance, no rotational cease was observed in the movement between navicular and cuboid, calcaneocuboid joint, or cuneonavicular joint.

**Conclusion:**

This study described foot bone motion using a biomechanically near-physiological gait simulator with 6 DOF of the tibia. The kinematic data helps to clarify previous descriptions of several joint kinematics that are difficult to study in vivo. The methodology also provides a platform for researchers to explore more invasive foot biomechanics under dynamic and near-physiologic conditions.

## Introduction

Foot and ankle play an important role in bearing weight and managing normal daily activities such as walking. Given the fact that biomechanics is essential for foot and ankle surgery, it is a necessity for us, orthopedists, to improve our understanding of foot biomechanics, especially the 3D kinematics of the foot bones during human gait.

Previous studies conducted for the measurement of 3D kinematics of the foot bones during gait can be classified into four categories [1–10], including skin marker-based motion capture system, fluoroscopy study, in vivo measurement with bone pins, and measurement through cadaver gait simulators. However, some limitations are found in previous research methods. First, the skin marker-based motion capture system is less accurate due to skin marker artifacts [1, 11]. Next, fluoroscopy study was found less accurate in bony motion description due to the low frequency of observation and human-based inaccuracy in registration [8, 9, 12]. Some others placed bone pins to provide valuable foot kinematic data in vivo models, but the highly invasive procedure made it hard to apply in living subjects [4, 13].

Previous scholars [4–6, 14–20] have made many types of cadaver gait simulators for foot biomechanical research, and some have used these machines to measure foot skeletal motion during gait directly. However, previous machines have their limitations, like unphysiological ground reaction force (GRF) [6, 14, 17, 19], low simulation gait speed [6, 20], and simplified tibia kinematics [6, 14, 18]. Whittaker made a special gait simulator which achieved 6 DOF of tibia control by rotating the force plate while keeping the foot static. Although this machine achieved six-degree freedom tibia control, this simulation way differed from traditional walking pattern. Furthermore, the peak vertical GRF was about 0.8 bodyweight (BW) during the gait, which was less than the normal value [21].

We have developed a cadaveric gait simulator with 6 DOF of tibia in a physiological way that moved the cadaver foot while keeping the force plate static. Furthermore, the peak of vertical GRF achieved physiological value ranging from 1.1 BW and 1.3 BW [22, 23]. The aim of this work was to study the foot bony motion and joints move in a gait cycle with this foot and ankle gait simulator.

## Method

### Materials

This study was approved by the Ethics Committee of our hospital. Six right-sided specimens were from three male and three female donators, whose death ages ranged from 45 to 69 years old with mixed death histories as shown in Table 1. None of the donators had evidence of prior foot surgery.

**Table 1:** Basic information of cadaveric feet

Number	Gender	Age	Height (cm)	Weight (kg)
1	Male	65	160	60
2	Male	69	167	85
3	Female	63	177	80
4	Female	48	178	72
5	Male	56	168	60
6	Female	52	161	54
Mean		58.8	168.5	68.5

These specimens were fresh frozen cadavers, defrosted overnight for the experiment. And they were dissected to provide access to leg tendons, but all structures remained intact below the malleoli. The nine tendons in the foot were divided into four bundles, including anterior group comprised of tibialis anterior, extensor hallucis longus, and extensor digitorum longus. The posterior bundle was Achilles tendon. Medial group comprised of tibialis posterior, flexor hallucis longus, and flexor digitorum longus. The lateral group comprised of peroneus brevis and peroneus longus. The muscle tissue of above nine tendons was dissected, but the tendon tissue remained intact.

**Fig. 1:**
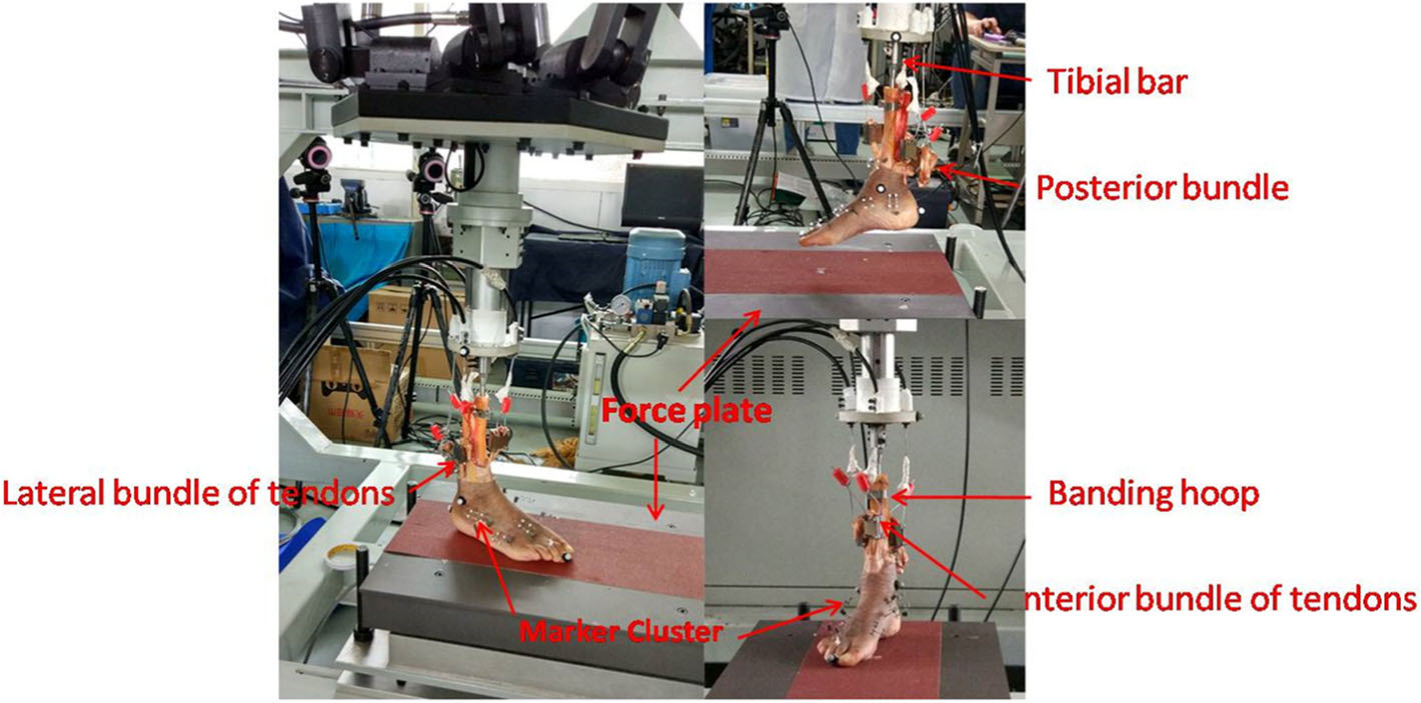
Cadaver foot connecting with the machine

Artificial muscle forces were generated by four motors, which connected to each of four groups of tendons through custom-made clamps, as shown in Fig. 1. The tibia was sawed off 250 mm upper from pelma when ankle was in a neutral position, and a 1.27-cm diameter drill was used to hollow out the tibia intramedullary canal. We inserted the metal bar into the tibia intramedullary canal and drilled two screws to fix the bar and tibia together. Another end of the bar was fixed in the simulator machine’s tibia control place with screws. A band hoop was used to tie tibia and fibula together.

Each of tibia, calcaneus, cuboid, navicular, medial cuneiform, and 1-3-5 metatarsals were drilled with 1.6 mm Kirschner wires for supporting markers, as shown in Fig. 1. A combination of the real markers and virtual markers (generated from static standing trial) was used to define the *x*-axis (medial/lateral), *y*-axis (anterior/posterior), and *z*-axis (vertical axis) of the local coordinate frames for each bone. When the foot was weight-bearing and static, the local frames for each bone were fit with the axes of the global frame, and the virtual markers were generated. The midline of the foot was aligned parallel to the *y* (anterior/posterior) axis of the global coordinate system and the tibia vertical.

**Fig. 2:**
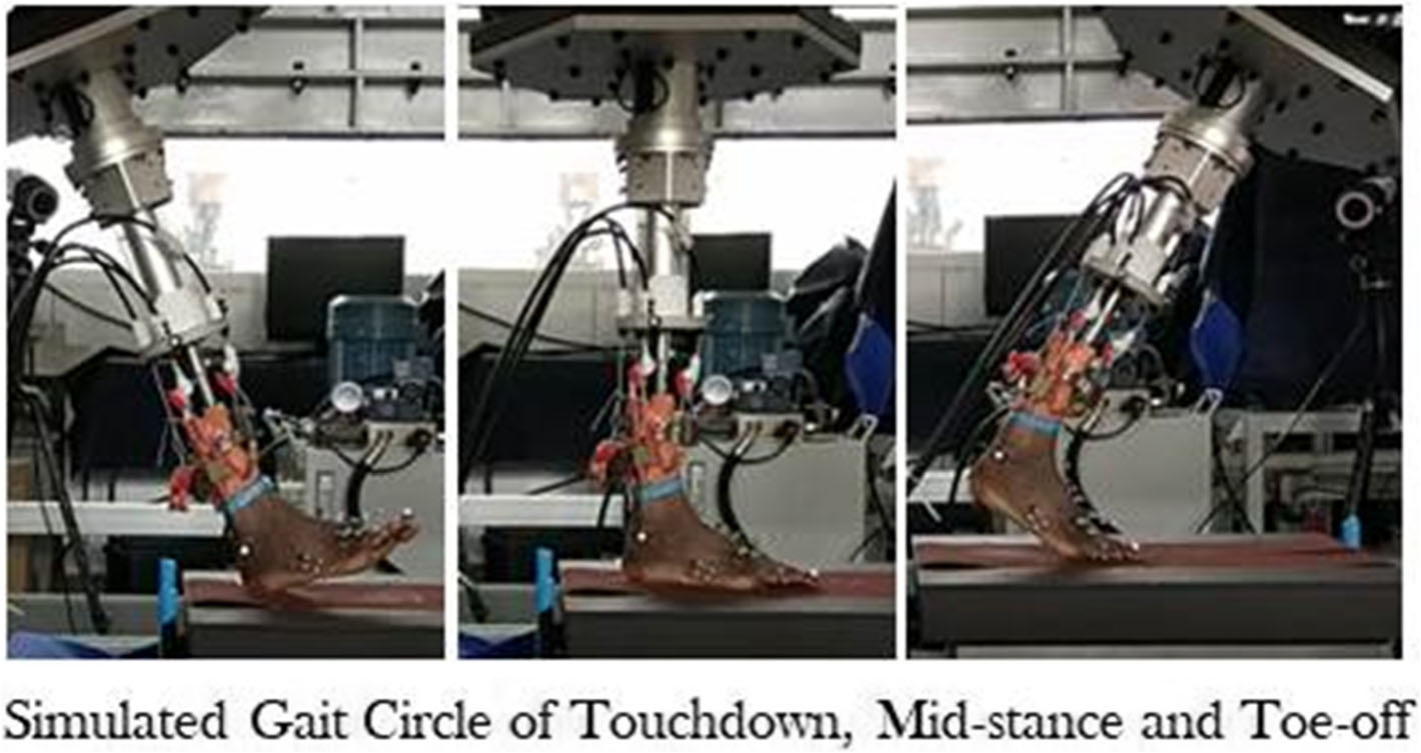
Three pictures showed the three state of gait (touchdown, midstance, and toe-off)

The simulation process started at heel contact and stopped when only toes were on the ground, and the metatarsal phalangeal joint was close to maximal dorsiflexion, as shown in Fig. 2. We collected movements of four joints (the cuneonavicular joint, the first tarsometatarsal joint, the calcaneocuboid joint, the fifth tarsometatarsal joint) relative motions of adjacent bones (including cuboid to navicular and calcaneus to tibia) during six gait simulation progress of each foot cadaver. The range of motions (ROMs) between adjacent bones in 3D was recorded, and the kinematic patterns were determined during the walking process.

The cadaveric gait simulator had another six actuating motors to control tibia to achieve a six-degree freedom parallel mechanism, including the direction of up-down, anterior-posterior, medial-lateral, and rotation in sagittal, in coronal, and in the transverse plane. For the control system, we used closed-loop control and iterative learning control to have a more real gait cycle. Lower limb cadaveric gait simulators aim to reproduce a normative vertical ground reaction force (vGRF) in vitro. During the cadaver gait simulations, the operator uses their expert knowledge of lower limb muscle and joint function to make educated trial-and-error guesses as to which muscle or kinematic input should be adjusted to achieve the desired vGRF. Closed-loop feedback control of the vGRF would likely improve the in vitro vGRF tracking accuracy and reduce the number of preliminary tuning simulations necessary to achieve vGRF tracking [17]. An iterative learning mechanism was to optimize the desired position trajectory of the hydraulic cylinder which controls the movement of the tibia. After a gait simulation trial, the recorded cylinder loading force would be analyzed, and the desired position trajectory of the cylinder would be adjusted by an iterative learning mechanism. Then, the optimized trajectory would be saved and utilized in the next iteration. This process would be repeated until the tibia loading force could converge to the target curve through several iterations [23].

Ground reaction forces were synchronously collected using a force plate system of 1000 Hz (FP4060-15-2000Bertec, USA), and kinematic data was recorded (100 Hz) using seven cameras (Qualisys (Mocap Camera–Miqus M3) Motion Capture Systems, Sweden) which were positioned close to the specimens. For each tested bone, the local coordinate system was parallel to the global coordinate system (*x*-axis: medial/lateral, *y*-axis: anterior/posterior, *z*-axis: vertical axis) when the foot was load-bearing. Furthermore, the foot midline was parallel to the *y*-axis, and the tibia was vertical. Simultaneously, the initial virtual marker cluster was also defined. In the subsequent trials, the position and orientation of each local coordinate system can be calculated by combining the real marker cluster and the initial virtual marker cluster.

## Result

There was a total of 6 cadaver feet, and every cadaver had 6 times of gait simulation in this experiment. During 36 (6 × 6) times of gait simulations, the mean value and the standard deviation (SD) of the total ROMs of the relative motions of two pairs of bone blocks (cuboid to navicular and calcaneus to the tibia) and four joints (the medial cuneonavicular joint, the first tarsometatarsal joint, the calcaneocuboid joint, and the fifth tarsometatarsal joint) were calculated on the sagittal, coronal, and transverse planes respectively, as shown in Table 2.

**Table 2:** Foot joint motion of three planes during simulated gait

Anatomical joint rotation angel (°)	Data source	Sagittal plane average ROM ± SD	Coronal plane average ROM ± SD	Transverse plane average ROM ± SD
Nav-Medcunif	Simulated results	7.4 ± 3.8	6.6 ± 2.0	5.4 ± 2.6
Medcunif-Met1	Simulated results	4.0 ± 1.5	6.6 ± 3.4	4.8 ± 2.3
Calc-Cub	Simulated results	6.7 ± 4.0	9.9 ± 4.7	7.5 ± 3.8
Cub-Nav	Simulated results	4.7 ± 1.8	6.1 ± 1.8	6.9 ± 0.7
Cub-Met5	Simulated results	9.1 ± 3.6	7.2 ± 2.5	6.8 ± 2.3
Calc-Tibia	Simulated results	27.1 ± 1.8	7.0 ± 1.6	8.2 ± 3.0

*Nav* navicular, *Medcunf* medial cuneiform, *Met1* the first metatarsal, *Calc* calcaneus, *Cub* cuboid, *Met5* the fifth metatarsal

**Fig. 3:**
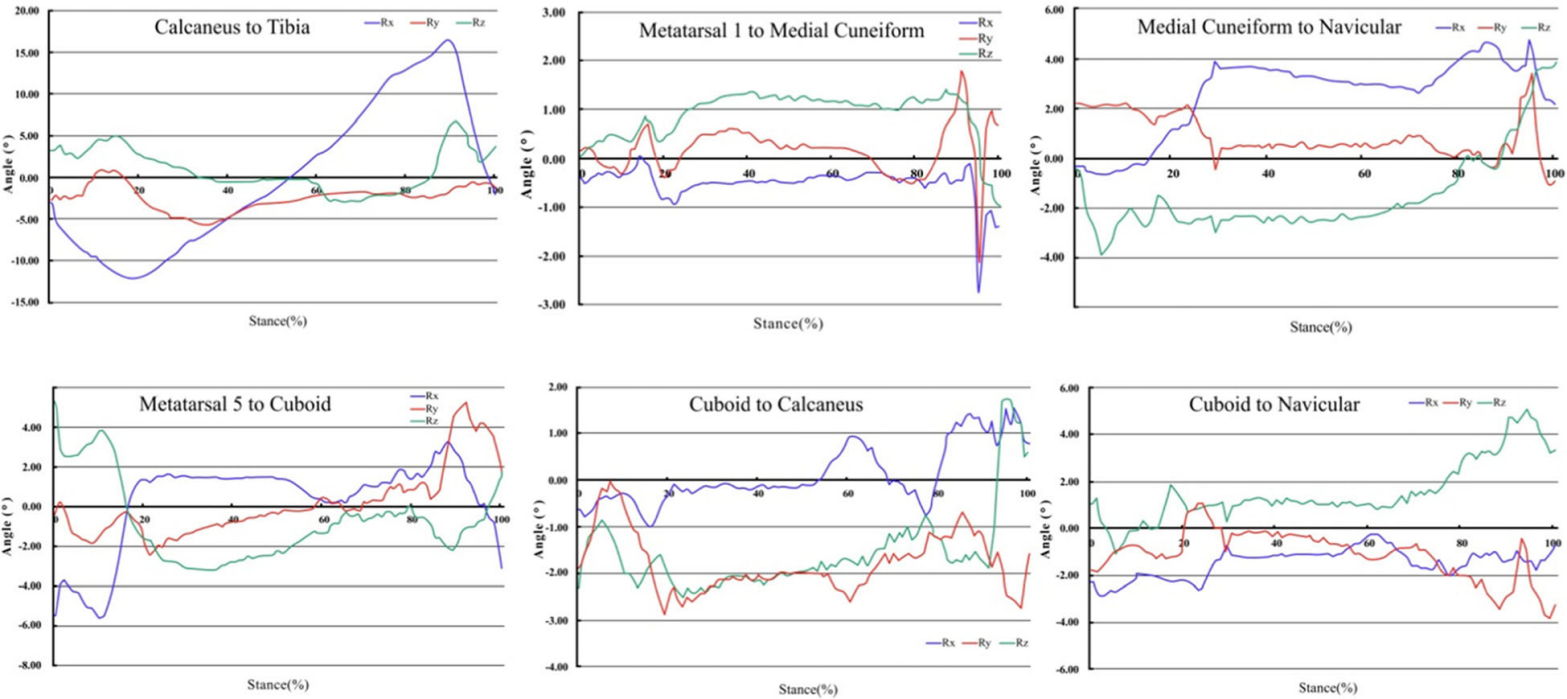
The mean movement of four joints and two sets of adjacent bones of six cadavers during simulated gait. Rx represents sagittal movement; more positive values are dorsiflexion; more negative values are plantar flexion. Ry represents coronal movement; more positive values are eversion; more negative values are inversion. Rz represents transverse movement; more positive values are external rotation; more negative values are internal rotation

In the sagittal plane, the medial cuneonavicular joint, the first tarsometatarsal joint, the calcaneocuboid joint, and the fifth tarsometatarsal joint all dorsiflexed from heel-strike followed by plantar flexion. The cuboid respect to navicular kept constant during heel-strike, then began to evert at gait phase 25%, and kept constant afterward. In the coronal plane, cuboid to calcaneus everted at heel-strike followed by inversion, remained relatively constant during foot flat, and inverted at push-off stage. The first tarsometatarsal joint, the medial cuneonavicular joint, and the fifth tarsometatarsal joint were relatively constant during heel-strike and midstance but everted at the push-off. The cuboid respecting to navicular everted at heel-strike, and followed by inversion during midstance, then everted at the push-off stage. In the transverse plane, the first tarsometatarsal joint began to adduct at heel-strike, kept constant in the midstance, but adducted at the push-off stage. The medial cuneonavicular joint, the fifth tarsometatarsal joint, and the calcaneocuboid joint began to adduct at heel-strike, kept constant during foot flat, but abducted at push-off. The cuboid respect to navicular kept constant during heel-strike and foot flat but abducted during push-off, as shown in Fig. 3.

## Discussion

It is of great importance to quantify the bony motion of the foot and ankle during gait. This knowledge is the foundation to understand its normal and pathologic function, to determine finite element boundary of the foot, and to give guidance of prosthetic joint design and replacement. Cadaver gait simulator was developed for foot bony kinematics measurement, including Whittaker et al.’s [21] bony motion measurement via a gait simulator, which was considered as the most advanced gait simulator. Although Baxter et al. [7] used their gait simulator constructed in the same way as Eric C. Whittaker et al.’s to assessed ankle, subtalar, and talonavicular kinematics, they did not study facet joints in the foot. Therefore, we compared our gait simulated results with Lundgren’s results and Whittaker’s results, as shown in Table 3.

**Table 3:** Foot joint motion of three planes during simulated gait with a comparison with previous results

Anatomical joint rotation angel (°)	Data source	Sagittal plane average ROM ± SD	Coronal plane average ROM ± SD	Transverse plane average ROM ± SD
Nav-Medcunif	Simulated results	7.4 ± 3.8	6.6 ± 2.0	5.4 ± 2.6
	Lundgren et al. [4]	11.5 ± 1.8	10.4 ± 6.3	6.2 ± 4.2
	Whittaker et al. [21]	12.2 ± 2.2	9.4 ± 2.4	5.8 ± 2.8
Medcunif-Met1	Simulated results	4.0 ± 1.5	6.6 ± 3.4	4.8 ± 2.3
	Lundgren et al. [4]	5.3 ± 2.0	5.4 ± 1.0	6.1 ± 1.1
	Whittaker et al. [21]	5.6 ± 2.3	8.5 ± 2.5	5.5 ± 2.1
Calc-Cub	Simulated results	6.7 ± 4.0	9.9 ± 4.7	7.5 ± 3.8
	Lundgren et al. [4]	9.7 ± 5.2	11.3 ± 3.9	8.1 ± 2.0
	Whittaker et al. [21]	8.8 ± 1.9	8.6 ± 0.5	7.5 ± 1.8
Cub-Nav	Simulated results	4.7 ± 1.8	6.1 ± 1.8	6.9 ± 0.7
	Lundgren et al. [4]	7.2 ± 2.4	8.8 ± 4.4	8.9 ± 4.3
	Whittaker et al. [21]	18.7 ± 9.4	4.9 ± 3.0	20.1 ± 11.1
Cub-Met5	Simulated results	9.1 ± 3.6	7.2 ± 2.5	6.8 ± 2.3
	Lundgren et al. [4]	13.3 ± 1.4	10.4 ± 3.7	9.8 ± 2.1
	Whittaker et al. [21]	12.3 ± 5.7	11.9 ± 1.3	9.1 ± 5.9
Calc-Tibia	Simulated results	27.1 ± 1.8	7.0 ± 1.6	8.2 ± 3.0
	Lundgren et al. [4] Whittaker et al. [21]	17.0 ± 2.1 23.6 ± 7.0	11.3 ± 3.5 9.2 ± 3.0	7.3 ± 2.4 10.7 ± 3.6

*Nav* navicular, *Medcunf* medial cuneiform, *Met1* the first metatarsal, *Calc* calcaneus, *Cub* cuboid, *Met5* the fifth metatarsal

In general, we found good agreement between our kinematic data and the data from in vivo studies by Lundgren, which is deemed as the gold standard. The total ROM reported here was within ± 1 SD (standard deviation) of the data reported by Lundgren et al. [4] for 13 out of 18 angles, while 15 of the 18 reported angles were within ± 2 SD of the data reported by Lundgren et al. [4].

Compared with the results of Whittaker et al. [21], our description of bone motion was similar to Whittaker’s work for the most part. However, we employed different simulation way from that of Whittaker et al. [21] and Baxter et al. [7]. Our machine achieved the goal of six freedom motion control of tibia more physiologically, while Eric C. Whittaker and Baxter J.R employed an unphysiological way by motivating ground and keeping cadavers still to reproduce gait. Furthermore, the longitudinal axis of cadavers in their simulating process was parallel to the floor instead of vertical as a human being walking way. Furthermore, the peak of GRF of our machine was equal to 1.1 BW and 1.3 BW, while Whittaker et al. [21] measured the kinematics of foot bony only in 75% bodyweight and Baxter J.R in 25% bodyweight. From our simulated results, we found that the movement during gait between cuboid and navicular (sag, 4.7°; corn, 6.1°; trans, 6.9°) was close to the Lundgren’s results (sag, 7.2°; corn, 8.8°; trans, 8.9°) but not as large as Whittaker’s [21] results(sag, 18.7°; corn, 4.9°; trans, 20.1°). And we assumed the gravity of cadaver itself and the insufficient GRF in simulation might influence the correction of the results in Eric C. Whittaker’s cadaver gait simulator.

There are several meaningful results from our work. First, we found that several joints in foot cannot be regard as a rigid body during gait process, especially the movements in intertarsal joints and tarsometatarsal joints, including the medial cuneonavicular joint (sag, 7.4°; corn, 6.6°; trans, 5.4°), the first tarsometatarsal joint (sag, 4.0°; corn, 6.6°; trans, 4.8°), the fifth tarsometatarsal joint (sag, 9.1°; corn, 7.2°; trans, 6.8°), the calcaneocuboid joint (sag, 6.7°; corn, 9.9°; trans, 7.5°), and navicular to cuboid (sag, 4.7; corn, 6.1; trans, 6.9). These joints provided movement of 11.4° in the sagittal plane, 13.6° in coronel plane, and 10.2° in the transverse plane during gait. These movements confirmed that they could not be regarded as a rigid body during gait [4], instead was an important complementary portion of foot motion during gait.

Second, we found that the medial column had less ROM than that in the lateral during gait. During the simulated walking stance, the joints in the medial column of foot remained constant, like the medial cuneonavicular joint and the first tarsometatarsal joint. However, the lateral column joints had more movement, like calcaneocuboid joint and the fifth metatarsal joint. Compared the motion of the first tarsometatarsal joint (sag, 4.0°; corn, 6.6°; trans, 4.8°), the fifth tarsometatarsal joint had a greater total ROM (sag, 9.1°; corn, 7.2°; trans, 6.8°), which suggested that the medial column had less ROM than the lateral during gait to provide a firm support for the weight. These findings gave instructions to our clinical surgery that it is unwise to do joint fusion or joint movement restriction in the fifth tarsometatarsal joint. It was reported by Davitt and Morgan [24] that two flat-foot patients suffered the fifth metatarsal fatigue fractures after lateral column lengthening surgery. We thought that too much lengthening in the lateral necessarily might restrict joint motion, which caused stress concentration. Nevertheless, it was admitted to do fusion in medial joints, like treating severe Lisfranc injury.

Last, our results did not support the midtarsal locking mechanism proposed by Mann [25] that the relative midtarsal bones motion would cease to produce a rigid foot, which could effectively propel bodyweight during the later portion of walking stance. However, our in vitro kinematic results did not support the existence of the midtarsal locking mechanism during the stance phase. From Fig. 3, no rotational cease was observed in the calcaneocuboid joint and the medial cuneonavicular joint, as well as in the movement between navicular and cuboid during the later portion of stance as proposed by the precious pocking mechanism. Furthermore, we found greater rotation in the latter portion of the stance than early portion. Challenge to the traditional locking mechanism had also been reported by Okita et al. [26] and Chen et al. [12]. They analyzed the midtarsal joint motion through a custom-made cadaveric gait simulator and fluoroscopic 3D–2D registration technique, respectively, and found the same phenomenon.

There are some limitations in the simulator, such as the reduced simulation velocity, faster increasing speed of the first peak of vertical GRF. And as other simulators machines, our cadaveric model did not simulate the intrinsic musculature force of the foot, which may be the reason why the results of bony motion were not correct enough compared to in vivo studies. Besides, some joints like subtalar joint and the first metatarsophalangeal joint were not included in the current study.

## Conclusion

This study described foot bone motion using a biomechanically near-physical gait simulator of six-degree freedom of tibia. Our kinematic data generally agreed with invasive in vivo research and provided a realistic description of bony motion for an in vitro model. The kinematic data helped to clarify previous descriptions of the function of several joints that were difficult to study in vivo.

## Data Availability

All data generated or analyzed during this study are included in this published article.

## Abbreviations

DOF: Degrees of freedom
3D kinematics of tibia: Three dimensional kinematics of tibia
GRF: Ground reaction force
BW: Bodyweight
ROMs: Range of motions
Nav: Navicular
Medcunf: Medial cuneiform
Met1: The first metatarsal
Calc: Calcaneus
Cub: Cuboid
Met5: The fifth metatarsal
SD: Standard deviation
